# Asymmetric Total Syntheses of Two 3-Acyl-5,6-dihydro-2*H*-pyrones: (*R*)-Podoblastin-S and (*R*)-Lachnelluloic Acid with Verification of the Absolute Configuration of (−)-Lachnelluloic Acid

**DOI:** 10.3390/molecules22010069

**Published:** 2017-01-01

**Authors:** Tetsuya Fujiwara, Takeshi Tsutsumi, Kohei Nakata, Hidefumi Nakatsuji, Yoo Tanabe

**Affiliations:** Department of Chemistry, School of Science and Technology, Kwansei Gakuin University, Sanda, Hyogo 669-1337, Japan; tt_fujiwara@jp.daicel.com (T.F.); dzx10859@kwansei.ac.jp (T.T.); dtp68975@kwansei.ac.jp (K.N.)

**Keywords:** asymmetric Mukaiyama aldol reaction, asymmetric total synthesis, natural fungicide, 3-acyl-5,6-dihydro-2*H*-pyrones, 1,3-bis(trimethylsiloxy)diene, podoblastin, lachnelluloic acid, alternaric acid

## Abstract

Expedient asymmetric total syntheses of both (*R*)-podoblastin-S and (*R*)-lachnelluloic acid, representative of natural 3-acyl-5,6-dihydro-2*H*-pyran-2-ones, were performed. Compared with the reported total synthesis of (*R*)-podoblastin-S (14 steps, overall 5% yield), the present study was achieved in only five steps in an overall 40% yield and with 98% ee (HPLC analysis). In a similar strategy, the first asymmetric total synthesis of the relevant (*R*)-lachnelluloic acid was achieved in an overall 40% yield with 98% ee (HPLC analysis). The crucial step utilized readily accessible and reliable Soriente and Scettri’s Ti(O*i*Pr)_4_/(*S*)-BINOL‒catalyzed asymmetric Mukaiyama aldol addition of 1,3-bis(trimethylsiloxy)diene, derived from ethyl acetoacetate with *n*-butanal for (*R*)-podoblastin-S and *n*-pentanal for (*R*)-lachnelluloic acid. With the comparison of the specific rotation values between the natural product and the synthetic specimen, the hitherto unknown absolute configuration at the C(6) position of (−)-lachnelluloic acid was unambiguously elucidated as 6*R*.

## 1. Introduction

**Figure 1:**
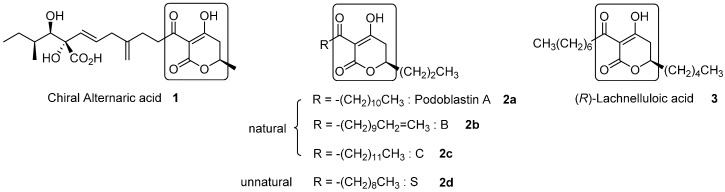
All reported natural products (**1**, **2a**–**c**, **3**) and the synthetic analogue (**2d**) possessing the 3-acyl-5,6-dihydro-2*H*-pyran-2-one structure.

3-Acyl-5,6-dihydro-2*H*-pyran-2-one is a unique heterocyclic molecule with a tricarbonyl moiety on the C(3)-carbon, which is found in natural products [1]. Figure 1 depicts all three natural products possessing the 3-acyl-5,6-dihydro-2*H*-pyran-2-one structure. Alternaric acid (**1**) is the most representative phytotoxic and antifungal compound isolated from *Alternaria solani* [2]. The unique and exquisite structure, with three contiguous chiral centers and non-conjugated dienes, renders this compound a noticeable synthetic target. The first total synthesis of chiral alternaric acid (**1**) was achieved by Ichihara’s group [3,4], and a formal synthesis was later performed by Trost’s group [5]. Our asymmetric total synthesis of **1** involves asymmetric Ti-Claisen condensation as a crucial step for the synthesis of elaborated left side chain [6]. In addition, very recently, we performed relevant asymmetric total synthesis of azaspirene, a unique hetero-spirocylic γ-lactam-type antibiotic, utilizing Ti-Claisen condensation and Ti-mediated direct aldol addition [7].

Natural (*R*)-podoblastins A–C (**2a**–**2c**), which exhibit anti-fungal activity against rice-blast disease, were isolated from *Podophyllum peltatum* L. by Sumitomo’s group [8]. Because the isolated products (**2a**–**2c**) were comprised of inseparable mixtures, accurate structural determination was required, in due course, via independent total syntheses of racemic compounds **2a**–**2c**. The obtained outcome unambiguously clarified that the naturally occurring specimens were made up of mixtures of A, B, and C (**2a**:**2b**:**2c** = 32:50:18) [8,9]. After the first synthesis of racemates **2a**–**2c** by one of the authors (Y.T.) [10], Takei’s group reported a second synthesis of podoblastin-S utilizing the unique 1,3-dipolar cycloaddition between acetylenecarboxylate and nitrile oxide [11].

This background led us to investigate the asymmetric total syntheses of a couple of chiral 3-acyl-5,6-dihydro-2*H*-pyrones, i.e., unnatural (*R*)-podoblastin-S (**2d**) [10] and natural (*R*)-lachnelluloic acid (**3**) [12], using a Ti-mediated reaction. The present two syntheses utilize efficient Ti(O*i*Pr)_4_/(*S*)-BINOL‒catalyzed asymmetric Mukaiyama aldol addition as a crucial step, developed by Soriente and Scettri’s group [13,14,15,16].

## 2. Results and Discussion

**Figure 2:**
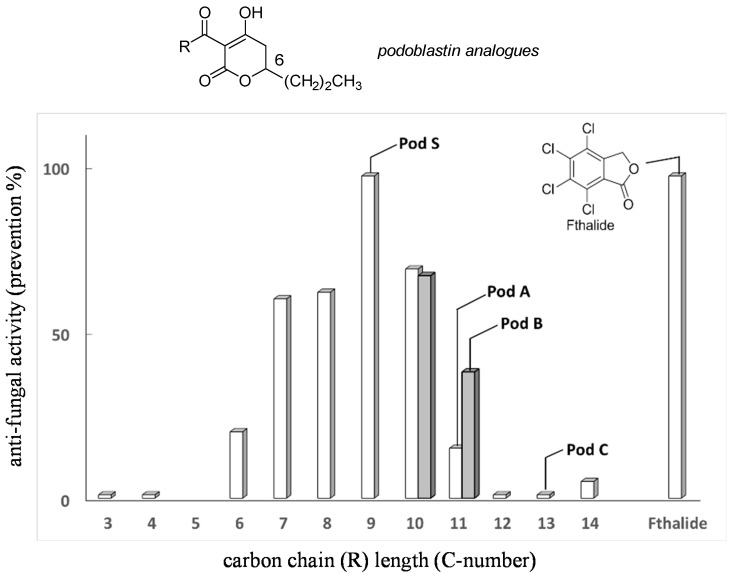
Relative activity of racemic podoblastin analogues with variations of C-3 acyl carbon long chain (*R*) length (assay method is described in Experimental Section. Cf. Shadowed prisms (C10 and C11) indicate the analogues possessing terminal double bond in R group.

Screening of synthetic racemate analogues of natural (*R*)-podoblastins **2a**–**2c** was carried out by one of the authors (Y.T.). This optimization, by changing the carbon long chain length of the 3-acyl moiety by settling the substituent in the 6-position as the *n*-Pr group, revealed that unnatural podoblastin-S (**2d**) had two- to three-fold stronger anti-fungal activity [12], using Fthalide as a representative fungicide reference [10] (Figure 2). On the other hand, the terminal double bond, as exemplified by podoblastin B, was found to be unimportant for anti-fungal activity. Thus, we selected asymmetric synthesis of (*R*)-podoblastin-S.

The first and sole chiral synthesis of (*R*)-podoblastin-S (**2d**) was performed by Ichimoto’s group, starting from (*S*)-1,3-dioxolane-4-methanol, a highly expensive chiral synthon, through 14 steps with an overall yield of 5% [17]. Our synthesis of **2d** involved a catalytic asymmetric Mukaiyama aldol addition as a crucial step, and was completed in a total of five steps. Moreover, we performed the first asymmetric total synthesis of (*R*)-lachnelluloic acid (**3**) containing the same 3-acyl-5,6-dihydro-2*H*-pyran-2-one structure as is in podoblastins. The unknown absolute configuration of natural (‒)-lachnelluloic acid (**3**) was unambiguously verified as (*R*), based on our synthetic strategy.

**Scheme 1:**
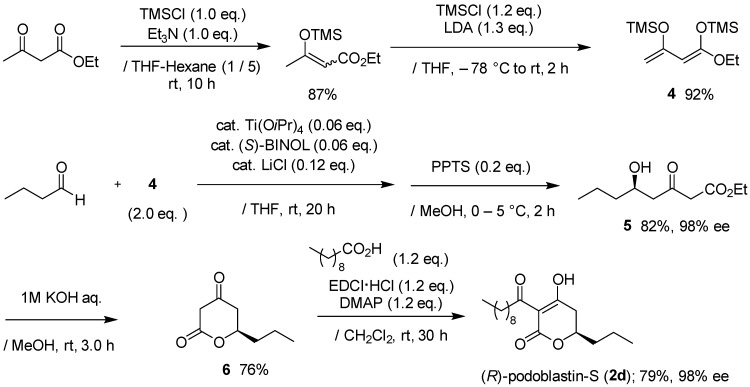
Asymmetric total synthesis of (*R*)-podoblasitin-S (**2d**).

As depicted in Scheme 1, our total synthesis started with the preparation of 1,3-bis(trimethylsiloxy)diene (Chan’s diene) **4**, which was obtained from ethyl acetoacetate in two steps in an 80% yield, according to the reported procedure [18,19,20,21]. Readily-accessible Soriente and Scettri’s Ti(O*i*Pr)_4_/(*S*)-BINOL‒catalyzed asymmetric Mukaiyama aldol addition of diene **4** with *n*CH_3_(CH_2_)_2_CHO successfully produced the desired (*R*)-δ-hydroxy-β-ketoester key intermediate **5** in an 82% yield with an excellent 98% ee (HPLC analysis, ESI). Note that the yield was slightly superior when using ethyl 1,3-bis(trimethylsiloxy)diene compared with other alkyl 1,3-bis(trimethylsiloxy)dienes, although the reason for this is unclear at present (Table 1).

Conventional KOH-hydrolysis of **5** and subsequent acid-catalyzed lactone formation afforded the desired (*R*)-5,6-dihydro-2*H*-pyran-2-one **6** in a 76% yield. For the *C*-acylation step, we adopted a mild and direct method, utilizing EDCI reagent [6], rather than indirect *O*-acylation and successive Fries-type rearrangement [9,15]. Thus, the final EDCI-mediated *C*-acylation of **6** with decanoic acid provided (*R*)-podoblastin-S (**2d**) in a 79% yield with a 98% ee (HPLC analysis, ESI). Consequently, the present total synthesis was performed in only five steps and in an overall 40% yield with 98% ee. Compared with the reported total synthesis [17], the overall yield and efficiency were improved remarkably.

Encouraged by the successful outcome, we next focused our attention on the relevant and first asymmetric total synthesis of (−)-lachnelluloic acid (**3**), which is also a natural anti-fungal product isolated from *Lachnellula fuscosanguinea* (Rehm) Dennis, as disclosed by Ayer and Villar [12]. (−)-Lachnelluloic acid (**3**) exhibits specific antagonistic activity against Dutch elm disease [22]. The first total synthesis of the racemic form of **3** was achieved by Ayer and Villar [9]. Later, a formal synthesis of racemate **3** was reported by Mineeva [23].

**Scheme 2:**
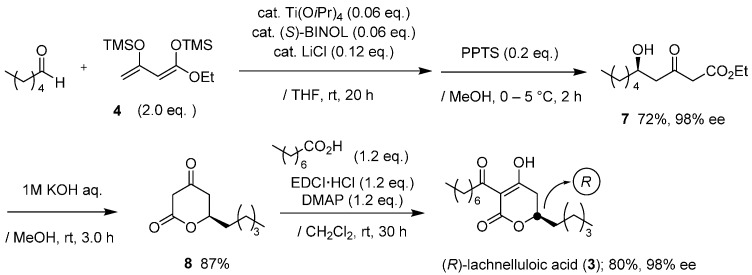
Asymmetric total synthesis of (*R*)-(−)-lachnelluloic acid (**3**).

Synthesis for chiral form of **3** was achieved in a similar method for podoblastin-S (Scheme 2). The reaction of **4** with *n*CH_3_(CH_2_)_3_CHO proceeded smoothly to give (*R*)-δ-hydroxy-β-ketoester adduct **7** in a 72% yield with an excellent 98% ee (HPLC analysis, ESI). Hydrolysis of **7**, followed by lactone formation, afforded the corresponding (*R*)-5,6-dihydro-2*H*-pyran-2-one **8** in an 87% yield. Final EDCI-mediated *C*-acylation of **8** with octanoic acid provided (*R*)-lachnelluloic acid (**3**) in an overall 40% yield with 98% ee (HPLC analysis, ESI. This asymmetric Mukaiyama aldol addition using Ti(O*i*Pr)_4_/(*S*)-BINOL consistently produces the (*R*)-aldol adduct [10]. Eventually, through the comparison of the specific rotation values between natural product **3** (${\left[\alpha \right]}_{D}^{25}$ ‒26.6 (*c* 10, MeOH)) [9] and synthetic specimen **3** (${\left[\alpha \right]}_{D}^{25}$ ‒24.4 (*c* 1, MeOH)), the configuration at the C(6) position in **3** was rigorously deduced as (*R*).

**Scheme 3:**
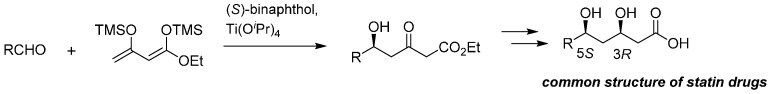
Synthesis of statin drugs utilizing the asymmetric Mukaiyama aldol addition with alkyl 1,3-bis(trimethylsiloxy)diene **4** as the key step.

Notably, the present strategy was applied as a promising process route of the key common component of well-known HMG-CoA reductase inhibitors (statin drugs) [24], such as pravastatin, simvastatin, atorvastatin, and pitavastatin (Scheme 3).

## 3. Materials and Methods

### 3.1. General

All reactions were carried out in oven-dried glassware under an argon atmosphere. Flash column chromatography was performed with silica gel Merck 60 (230–400 mesh ASTM, Darmstadt, Germany). TLC analysis was performed on 0.25 mm Silicagel Merck 60 F_254_ plates. Melting points were determined on a hot stage microscope apparatus (AS ONE, ATM-01, Osaka, Japan and were uncorrected. NMR spectra were recorded on a JEOL DELTA 300 (Tokyo, Japan) or JEOLRESONANCE ECX-500 spectrometer (Tokyo, Japan), operating at 300 MHz or 500 MHz for ^1^H-NMR and 75 MHz 120 MHz for ^13^C-NMR. Chemical shifts (δ ppm) in CDCl_3_ were reported downfield from TMS (= 0) for ^1^H-NMR. For ^13^C-NMR, chemical shifts were reported in the scale relative to CDCl_3_ (77.00 ppm) as an internal reference.IR Spectra were recorded on a JASCO FT/IR-5300 spectrophotometer (Tokyo, Japan). Mass spectra were measured on a JEOL JMS-T100LC spectrometer (Tokyo, Japan). HPLC data were obtained on a SHIMADZU HPLC system (consisting of the following: LC-20AT, CMB20A, CTO-20AC, and detector SPD-20A measured at 254 nm, Kyoto, Japan) using Daicel Chiracel AD-H or Ad-3 column (25 cm) at 25 °C. Optical rotations were measured on a JASCO DIP-370 (Na lamp, 589 nm, Tokyo, Japan).

### 3.2. 4-Ethoxy-2,2,8,8-tetramethyl-6-methylene-3,7-dioxa-2,8-disilanon-4-ene *(**4**)*

TMSCl (13.0 mL, 0.10 mol) was added to a stirred solution of ethyl acetoacetate (13.0 g, 0.10 mol) in THF–hexane (1 / 5, 150 mL) at 0–5 °C under an Ar atmosphere. After being stirred for 0.5 h, Et_3_N (14.0 mL, 0.10 mol) was added to the mixture, which was stirred at the same temperature for 0.5 h. The mixture was allowed to warm up to room temperature and the mixture was stirred for 14 h. The resulting mixture was reversely quenched with ice-water, which was extracted twice with hexane. The combined organic phase was washed with water, brine, dried (Na_2_SO_4_), and concentrated. The obtained crude oil was purified by distillation (bp 40–42 °C/3.0 kPa) to give the desired methyl 2-(trimethylsilyl)oxybut-2-enoate (17.6 g, 87%).

*n*BuLi (1.45 M in hexane, 18 mL, 26 mmol) was added to stirred solution of *i*Pr_2_NH (3.7 mL, 26 mmol) in THF (16 mL) at 0–5 °C under an Ar atmosphere, and the mixture was stirred for 5 min. The mixture was cooled down to −78 °C and ethyl 2-(trimethylsilyl)oxybut-2-enoate (4.05 g, 20 mmol) in THF (2.0 mL) was added dropwise over 3 min to the mixture, which was stirred at the same temperature for 0.5 h. TMSCl (3.0 mL, 26 mmol) in THF (2.0 mL) was added dropwise for 10 min to the mixture at the same temperature and the mixture was allowed to warm up to 0–5 °C over a period of 2 h. The mixture was concentrated using a rotary evaporator and filtered through Celite^®^(No. 503) using a glass filter, being washed with hexane (10 mL × 3). The filtrate was concentrated under reduced pressure to give the crude product **4** (5.03 g, 92%) [13,14], which was used for the next reaction without any purification.

Yellow oil; bp 52–55 °C/50 Pa; ^1^H-NMR (500 MHz, CDCl_3_): δ = 0.22 (s, 9H), 0.25 (s, 9H), 0.88 (t, *J* = 6.9 Hz, 3H), 3.77 (m, 2H), 3.90 (s, 1H), 4.13 (s, 1H), 4.46 (s, 1H); ^13^C-NMR (125 MHz, CDCl_3_): δ = 0.2, 0.4, 54.9, 77.6, 89.2, 153.3, 158.5; ν_max_ (neat) cm^−1^ 2961, 1649, 1443, 1391, 1250, 1196, 1090, 1015, 982, 835. ^1^H-NMR and ^13^C-NMR spectra: see supporting information.

### 3.3. Ethyl (R)-5-Hydroxy-3-oxooctanoate *(**5**)*

Preparation for Ti-BINOL solution: A suspension of Ti(O*i*Pr)_4_ (17.2 mg, 60 μmol) and (*S*)-BINOL (17.1 mg, 60 μmol) in THF (1.4 mL) was stirred at 20–25 °C under an Ar atmosphere for 20 min.

Asymmetric Mukaiyama aldol reaction: The obtained Ti-BINOL solution was added to a stirred suspension of butanal (72 mg, 1.0 mmol) and LiCl (5.1 mg, 120 μmol) in THF (1.6 mL) at 20–25 °C under an Ar atmosphere, followed by being stirred at the same temperature for 20 min. Diene **4** (549 mg, 2.0 mmol) in THF (1.0 mL) was added slowly to the mixture, which was stirred for 14 h. PPTS (50 mg, 0.20 mmol) in MeOH (2.0 mL) was added to the mixture, followed by being stirred at 0–5 °C for 2 h. The resulting mixture was quenched with sat. NaHCO_3_ aq., which was extracted twice with AcOEt. The combined organic phase was washed with water, brine, dried (Na_2_SO_4_), and concentrated. The obtained crude oil was purified by SiO_2_–column chromatography (hexane‒AcOEt = 5:1) to give the desired product **5** (170 mg, 84%).

Pale yellow oil; ${\left[\alpha \right]}_{D}^{25}$ −31.8 (*c* 1.0, CHCl_3_); ^1^H-NMR (500 MHz, CDCl_3_): δ = 0.93 (t, *J* = 7.3 Hz, 3H), 1.28–1.61 (m, 7H), 2.62-2.75 (m, 2H), 3.47 (s, 2H), 4.09 (m, 1H), 4.20 (q, 2H); ^13^C-NMR (125 MHz, CDCl_3_): δ = 13.8, 14.0, 18.6, 38.5, 49.6, 49.8, 61.4, 67.2, 166.9, 203.7; IR (neat): ν_max_ = 3502, 2957, 2874, 1742, 1711, 1651, 1437, 1321, 1263, 1150, 1009 cm^−1^; HRMS (ESI): *m/z* calcd for C_10_H_18_O_4_ [M + Na]^+^ 225.1103; found: 225.1101; >99% ee; HPLC analysis (Daicel, AD-H, flow rate 1.00 mL/min, solvent: hexane/EtOH = 20/1) t_R_(racemic) = 12.88 min and 19.52 min. t_R_[(*R*)-form] = 19.32 min. ^1^H-NMR and ^13^C-NMR spectra: see supporting information.

### 3.4. (R)-6-Propyldihydro-2H-pyran-2,4(3H)-dione *(**6**)*

(*R*)-Aldol adduct **5** (202 mg, 1.0 mmol) was added to a stirred 1M-KOH aq. solution (1.1 mL) at 0–5 °C under an Ar atmosphere and the mixture was stirred at 20–25 °C for 3 h. The resulting mixture was quenched with 1M-HCl aq., which was extracted twice with AcOEt. The combined organic phase was washed with water, brine, dried (Na_2_SO_4_), and concentrated. The obtained crude solid was washed with hexane (30 mL) to give the desired product **6** (121 mg, 77%).

Pale yellow crystals; mp 90–93 °C (lit. [17] mp 88–89 °C; ${\left[\alpha \right]}_{D}^{25}$ −82.8 (*c* 1.0, CHCl_3_)) (lit. [14] ${\left[\alpha \right]}_{D}^{18}$ −83.02 (*c* 1.69, CHCl_3_)); ^1^H-NMR (400 MHz, CDCl_3_): δ = 0.99 (t, *J* = 7.3 Hz, 3H), 1.42–1.72 (m, 3H), 1.79–1.88 (m, 1H), 2.47 (dd, *J* = 11.0 Hz, 18.3 Hz, 1H), 2.71 (dd, *J* = 2.8 Hz, 18.3 Hz, 1H), 3.43 (d, *J* = 18.8 Hz, 1H), 3.58 (d, *J* = 18.8 Hz, 1H), 4.62–4.68 (m, 1H); ^13^C-NMR (100 MHz, CDCl_3_): δ = 13.6, 18.0, 36.5, 43.5, 47.0, 75.3, 167.3, 200; IR (neat): ν_max_ = 2955, 2668, 1682, 1582, 1389, 1287, 1254, 1223, 1128, 1042, 878, 829 cm^−1^; HRMS (ESI): *m*/*z* calcd for C_8_H_12_O_3_ [M + Na]^+^ 179.0684; found: 179.0682. ^1^H-NMR and ^13^C-NMR spectra: see supporting information.

### 3.5. (R)-Podoblastin S (**2d**): (R)-3-Decanoyl-4-hydroxy-6-propyl-5,6-dihydro-2H-pyran-2-*one*

1-Ethyl-3-(3-dimethylaminopropyl)carbodiimide hydrochloride (EDCI·HCl) (230 mg, 1.2 mmol) was added to a stirred solution of (*R*)-pyrone **6** (156 mg, 1.0 mmol), decanoic acid (207 mg, 1.2 mmol), and DMAP (147 mg, 1.2 mmol) in CH_2_Cl_2_ (3.0 mL) at 0–5 °C under an Ar atmosphere, and the mixture was stirred for 30 h at 20–25 °C. The resulting mixture was quenched with 1M-HCl aq., which was extracted twice with CH_2_Cl_2_. The combined organic phase was washed with water, brine, dried (Na_2_SO_4_), and concentrated. The obtained crude oil was purified by SiO_2_–column chromatography (hexane‒AcOEt = 20:1) to give the desired product (245 mg, 79%).

Pale yellow crystals; mp 38–39 °C [lit. [17], 40.5–41.5 °C]; ${\left[\alpha \right]}_{D}^{25}$ −28.5 (*c* 1.0, CHCl_3_) [lit. [14], ${\left[\alpha \right]}_{D}^{22}$ −30.09 (*c* 1.19, CHCl_3_)]; ^1^H-NMR (500 MHz, CDCl_3_): δ = 0.87 (t, *J* = 6.7 Hz, 3H), 0.97 (t, *J* = 6.7 Hz, 3H), 1.19-1.83 (m, 18H), 2.55–2.70 (m, 2H), 2.96–3.09 (m, 2H), 4.34–4.50(m, 1H), 16.2 (s, 1H); ^13^C-NMR (125 MHz, CDCl_3_): δ = 13.7, 14.1, 17.9, 22.6, 25.0, 29.2, 29.30, 29.34, 29.4, 31.8, 36.7, 37.9, 38.5, 73.6, 103.1, 164.3, 195.2, 204.6; IR (neat): ν_max_ = 2924, 2855, 1713, 1557, 1464, 1273, 1238, 1063, 912 cm^−1^; HRMS (ESI): *m*/*z* calcd for C_18_H_30_O_4_ [M + H]^+^ 311.2222; found: 311.2209; 98% ee; HPLC analysis (Daicel, AD-H, flow rate 1.0 mL/min, solvent: hexane/EtOH = 20/1) t_R_(racemic) = 14.02 min and 16.52 min. t_R_[(*R*)-form] = 18.22 min. ^1^H-NMR and ^13^C-NMR spectra: see supporting information.

### 3.6. Ethyl (R)-5-hydroxy-3-oxodecanoate *(**7**)*

Preparation for Ti-BINOL solution: A suspension of Ti(O*i*Pr)_4_ (17.2 mg, 60 μmol) and (*S*)-BINOL (17.1 mg, 60 μmol) in THF (1.4 mL) was stirred at 20–25 °C under an Ar atmosphere for 20 min.

Asymmetric Mukaiyama aldol reaction: The obtained Ti-BINOL solution was added to a stirred suspension of hexanal (100 mg, 1.0 mmol) and LiCl (5.1 mg, 120 μmol) in THF (1.6 mL) at 20–25 °C under an Ar atmosphere, and the mixture was stirred at the same temperature for 0.5 h. Diene **4** (549 mg, 2.0 mmol) in THF (1.0 mL) was added slowly to the mixture, which was stirred for 14 h. PPTS (50 mg, 0.20 mmol) in MeOH (2.0 mL) was added to the mixture, followed by being stirred at 0–5 °C for 2 h. The resulting mixture was quenched with sat. NaHCO_3_ aq., which was extracted twice with AcOEt. The combined organic phase was washed with water, brine, dried (Na_2_SO_4_), and concentrated. The obtained crude oil was purified by SiO_2_–column chromatography (hexane-AcOEt = 5:1) to give the desired product **7** (163 mg, 71%).

Pale yellow oil; ${\left[\alpha \right]}_{D}^{25}$ −27.9 (*c* 1.0, CHCl_3_); ^1^H-NMR (500 MHz, CDCl_3_): δ = 0.89 (t, *J* = 7.5 Hz, 3H), 1.27–1.50 (m, 11H), 2.62–2.76 (m, 2H), 3.47 (s, 2H), 4.07 (m, 1H), 4.21 (q, *J* = 7.5 Hz, 2H); ^13^C-NMR (125 MHz, CDCl_3_): δ = 13.9, 14.0, 22.5, 25.0, 31.6, 36.4, 49.6, 49.8, 61.4, 67.5, 166.9, 203.7; IR (neat): ν_max_ = 3500, 2932, 2860, 1737, 1710, 1635, 1467, 1317, 1234, 1150, 1028 cm^−1^; HRMS (ESI): *m*/*z* calcd for C_12_H_22_O_4_ [M + Na]^+^ 253.1416; found: 253.1416; 98% ee; HPLC analysis (Daicel, AD-H, flow rate 1.00 mL/min, solvent: hexane/EtOH = 20/1) t_R_(racemic) = 11.52 min and 15.62 min. t_R_[(*R*)-form] = 15.61 min. ^1^H-NMR and ^13^C-NMR spectra: see supporting information.

### 3.7. (R)-6-Penthyldihydro-2H-pyran-2,4(3H)-dione *(**8**)*

(*R*)-Aldol adduct **7** (590 mg, 2.6 mmol) was added to a stirred 1M-KOH aq. solution (2.8 mL) at 0–5 °C under an Ar atmosphere and the mixture was stirred at 20–25 °C for 3 h. The resulting mixture was quenched with 1M-HCl aq., which was extracted twice with AcOEt. The combined organic phase was washed with water, brine, dried (Na_2_SO_4_), and concentrated. The obtained crude solid was washed with hexane (30 mL) to give the desired product **8** (417 mg, 87%).

Colorless crystals; mp 92–94 °C; ${\left[\alpha \right]}_{D}^{25}$ −71.4 (*c* 1.0, CHCl_3_); ^1^H-NMR (500 MHz, CDCl_3_): δ = 0.91 (t, *J* = 7.5 Hz, 3H), 1.33-1.55 (8H, m), 1.70 (1H, m), 1.83 (1H, m), 2.44–2.50 (dd, *J* = 11.5 Hz, 29.8 Hz, 1H), 2.69-2.73 (dd, *J* = 2.3 Hz, 20.6 Hz, 1H), 3.42 (d, *J* = 18.3 Hz, 1H), 3.55 (d, *J* = 18.9 Hz, 1H), 4.63 (m, 1H); ^13^C-NMR (125 MHz, CDCl_3_): δ = 13.8, 22.3, 24.3, 31.2, 34.4, 43.3, 46.9, 75.5, 167.5, 200.3; IR (neat): ν_max_ = 2953, 2859, 2669, 1672, 1581, 1389, 1283, 1237, 1211, 1128, 1180, 885, 736 cm^−1^; HRMS (ESI): *m*/*z* calcd for C_10_H_16_O_3_ [M + Na]^+^ 207.0997; found: 207.1001. ^1^H-NMR and ^13^C-NMR spectra: see supporting information.

### 3.8. (R)-Lachnelluoic acid *(**3**)*: (R)-3-Octanoyl-4-hydroxy-6-penthyl-5,6-dihydro-2H-pyran-2-*one*

EDCI·HCl (230 mg, 1.2 mmol) was added to a stirred suspension of (*R*)-pyrone **8** (184 mg, 1.0 mmol), octanoic acid (173 mg, 1.2 mmol), and DMAP (147 mg, 1.2 mmol) in CH_2_Cl_2_ (3.0 mL) at 0–5 °C under an Ar atmosphere, and the mixture was stirred for 30 h at 20–25°C. The resulting mixture was quenched with 1M-HCl aq., which was extracted twice with CH_2_Cl_2_. The combined organic phase was washed with water, brine, dried (Na_2_SO_4_), and concentrated. The obtained crude oil was purified by SiO_2_–column chromatography (hexane‒AcOEt = 25:1) to give the desired product (270 mg, 87%).

Colorless crystals: mp 43–45 °C [lit. [9] mp 39–40 °C]; ${\left[\alpha \right]}_{D}^{25}$ −24.4 (*c* 1.0, MeOH); (lit. [9] ${\left[\alpha \right]}_{D}^{25}$ −26.6 (*c* 10, MeOH)). ^1^H-NMR (500 MHz, CDCl_3_): δ = 0.88 (t, *J* = 6.9 Hz, 3H), 1.29–1.67 (m, 20H), 1.79 (m, 1H), 2.58–2.70 (m, 2H), 2.97–3.09 (m, 2H), 4.36–4.47 (m, 1H), 17.90 (s, 1H); ^13^C-NMR (125 MHz, CDCl_3_): δ = 13.9, 14.0, 22.4, 22.5, 24.3, 25.0, 29.0, 29.2, 31.4, 31.6, 34.5, 37.9, 38.4, 73.8, 103.1, 164.3, 195.1, 204.5; IR (neat): ν_max_ = 2955, 2928, 2859, 1715, 1557, 1456, 1406, 1259, 1067, 907, 729 cm^−1^; HRMS (ESI): *m*/*z* calcd for C_18_H_30_O_4_ [M + H]^+^ 311.2222; found: 311.2223; 98% ee. ^1^H-NMR and ^13^C-NMR spectra: see supporting information.

Assay method for podoblastin analogues: Definite amounts (100 ppm) of the testing sample, which was emulsified with Sorpol and water, were sprayed on rice plant (*Oryza sativa* L. var. Kinki No. 33, 2.5 th leaf stage). After 4 h, inoculation of *Pyricularia oryzae* was carried out by spraying a spore suspension contained ca. 10^7^ spores/mL to the test plant, then incubated at 28 °C with >95% humidity for 4 days.

## 4. Conclusions

We performed efficient total syntheses of both (*R*)-podoblastin-S and (*R*)-lachnelluloic acid in only five steps using a common synthetic strategy with excellent overall yields (each 40%) and enantiomeric excesses (each 98%). The absolute configuration of (−)-lachnelluloic acid was unambiguously elucidated as 6*R* by virtue of readily-accessible and reliable Soriente’s Ti(O*i*Pr)_4_/(*S*)-BINOL‒catalyzed asymmetric Mukaiyama aldol addition of 1,3-bis(trimethylsiloxy)diene, derived from ethyl acetoacetate. The present method provides practical and substrate-general synthetic access for the chiral building block, δ-hydroxy-β-ketoesters and 5,6-dihydro-2*H*-pyran-2-ones.

Eventually, we achieved asymmetric total syntheses of all three natural products comprising the 3-acyl-5,6-dihydro-2*H*-pyrone structure, alternaric acid (the preceding report [6]), (*R*)-podoblastin-S, and (*R*)-lachnelluloic acid (the present report), involving characteristic asymmetric Ti-mediated addition and condensation reactions as key steps.

## Figures and Tables

**Table 1:**
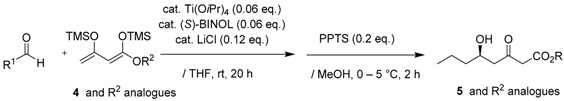
Ti(O*i*Pr)_4_/(*S*)-BINOL‒catalyzed asymmetric Mukaiyama aldol addition of diene **4** and its analogues with *n*CH_3_(CH_2_)_2_CHO and *n*CH_3_(CH_2_)_4_CHO.

Entry	R^1^	R^2^	Yield (%)	ee (%)
1	*n*CH_3_(CH_2_)_2_	Me	71	98
2		Et	84	>98
3		*i*Pr	82	98
4		*t*Bu	Complex mixtures	–
5	*n*CH_3_(CH_2_)_4_	Et	72	98

## Supplementary Materials

Copies of the ^1^H, ^13^C-NMR spectra for compounds **4**, **5**, **6**, **2d**, **7**, **8**, and **3** are available in the supplementary materials. The supplementary materials are available online at www.mdpi.com/1420-3049/22/1/69/s1.
