# Supplementary material for: Asymmetric Total Syntheses of Two 3-Acyl-5,6-dihydro-2H-pyrones: (R)-Podoblastin-S and (R)-Lachnelluloic Acid with Verification of the Absolute Configuration of (−)-Lachnelluloic Acid
Source: Molecules. 2017 Jan 1;22(1):69. doi: 10.3390/molecules22010069 (PMC6155883; doi:10.3390/molecules22010069)
Supplement: Supplementary file 1 [file molecules-22-00069-s001.pdf]

# Supplementary Materials: Asymmetric Total Syntheses of Two 3-Acyl-5,6-dihydro-2*H*-pyrones: (*R*)-Podoblastin-S and (*R*)-Lachnelluloic Acid with Verification of the Absolute Configuration of (-)-Lachnelluloic Acid

**Tetsuya Fujiwara, Takeshi Tsutsumi, Kohei Nakata, Hidefumi Nakatsuji and Yoo Tanabe**

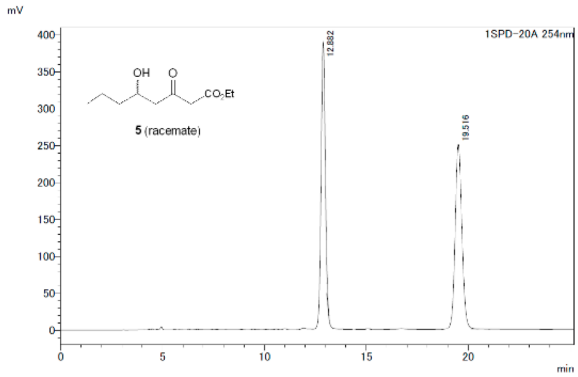


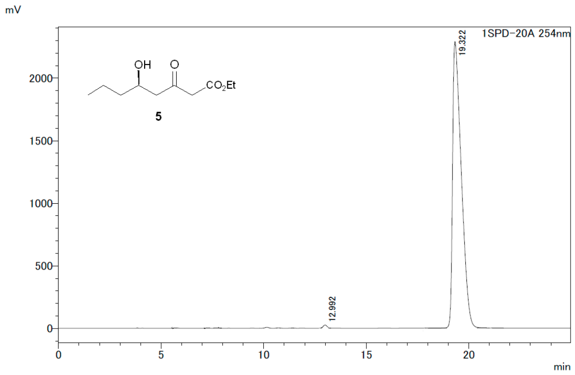


SPD-20A 254nm

| peak<br>ピーク# | retention time<br>保持時間 | area<br>面積 | height<br>高さ | %<br>濃度 |
|---|---|---|---|---|
| 1 | 12.992 | 300783 | 23734 | 0.443 |
| 2 | 19.322 | 67529431 | 2290441 | 99.557 |
| 合計 | | 67830213 | 2314175 | |

(A)

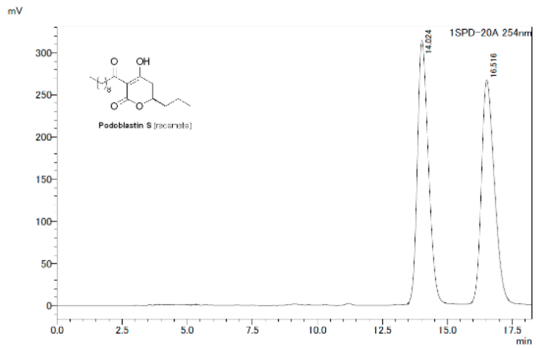


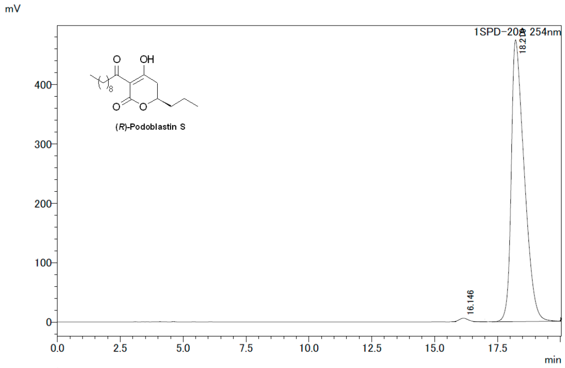


SPD-20A 254nm

| peak<br>ピーク# | retention time<br>保持時間 | area<br>面積 | height<br>高さ | %<br>濃度 |
|---|---|---|---|---|
| 1 | 16.146 | 164058 | 6149 | 0.952 |
| 2 | 18.219 | 17069134 | 474770 | 99.048 |
| 合計 | | 17233193 | 480919 | |

(B)

**Figure S1.** HPLC data of aldol adduct **5** (**A**) and podoblastin-S (**B**).

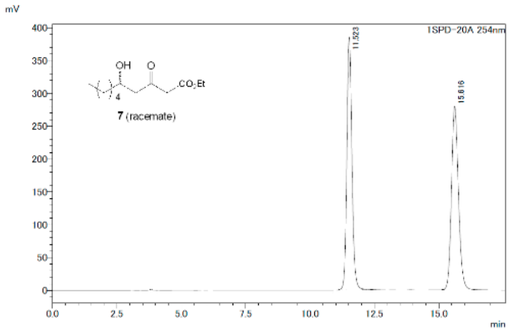


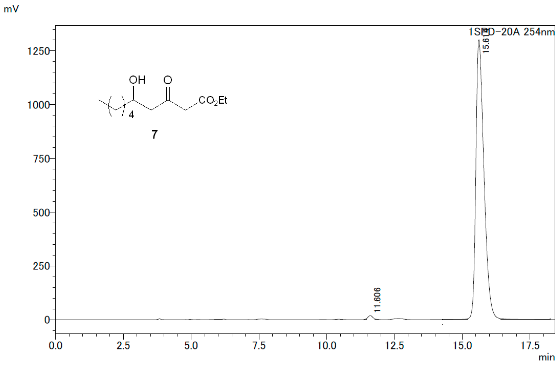


SPD-20A 254nm

| ピーク# (peak) | 保持時間 (retention time) | 面積 (area) | 高さ (height) | 濃度 (%) |
|---|---|---|---|---|
| 1 | 11.606 | 235199 | 18297 | 0.861 |
| 2 | 15.614 | 27085229 | 1300823 | 99.139 |
| 合計 | | 27320428 | 1319120 | |

**Figure S2.** HPLC data of aldol adduct **7**.

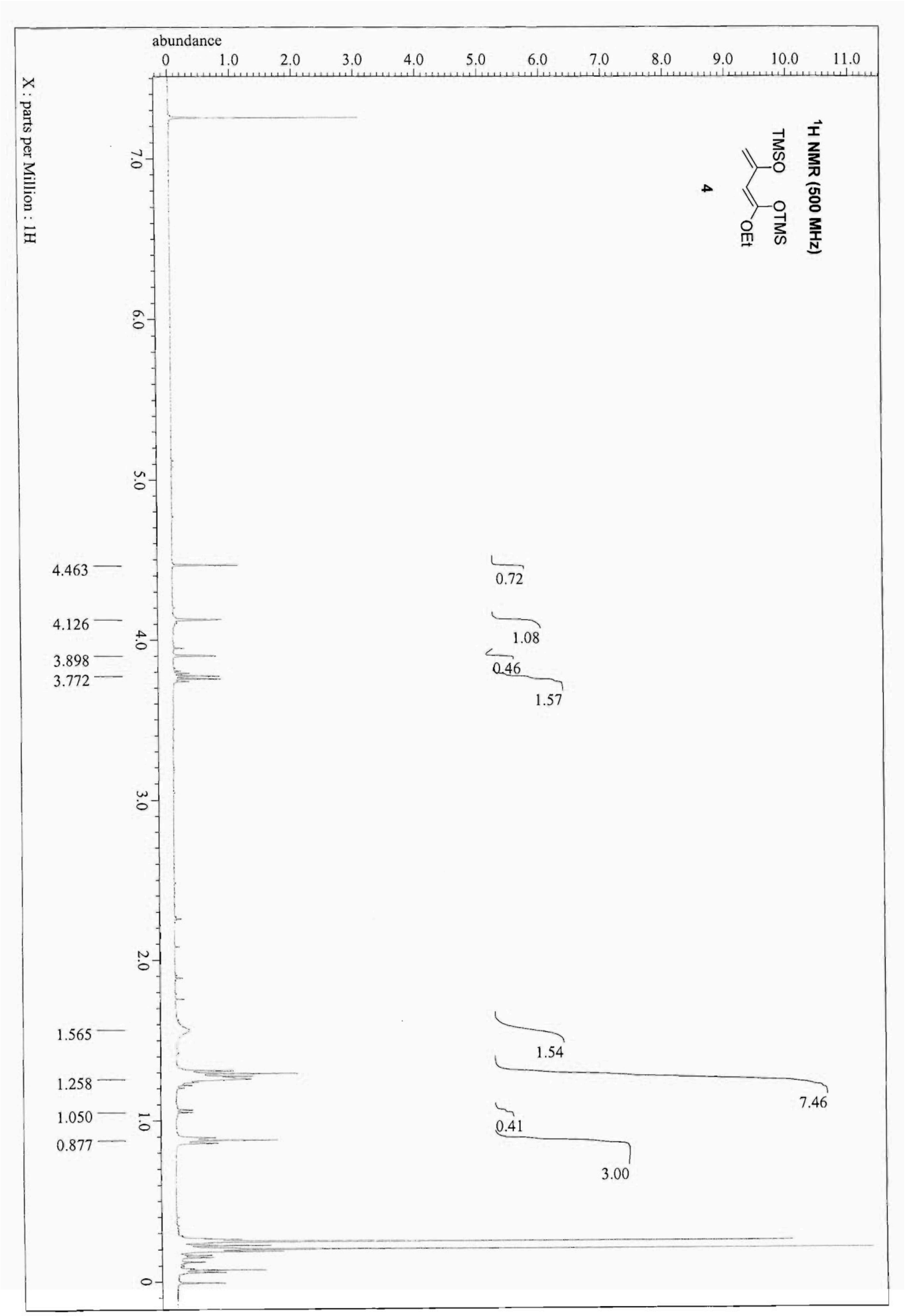


**Figure S3.** $^1$H-NMR chart of **4**.

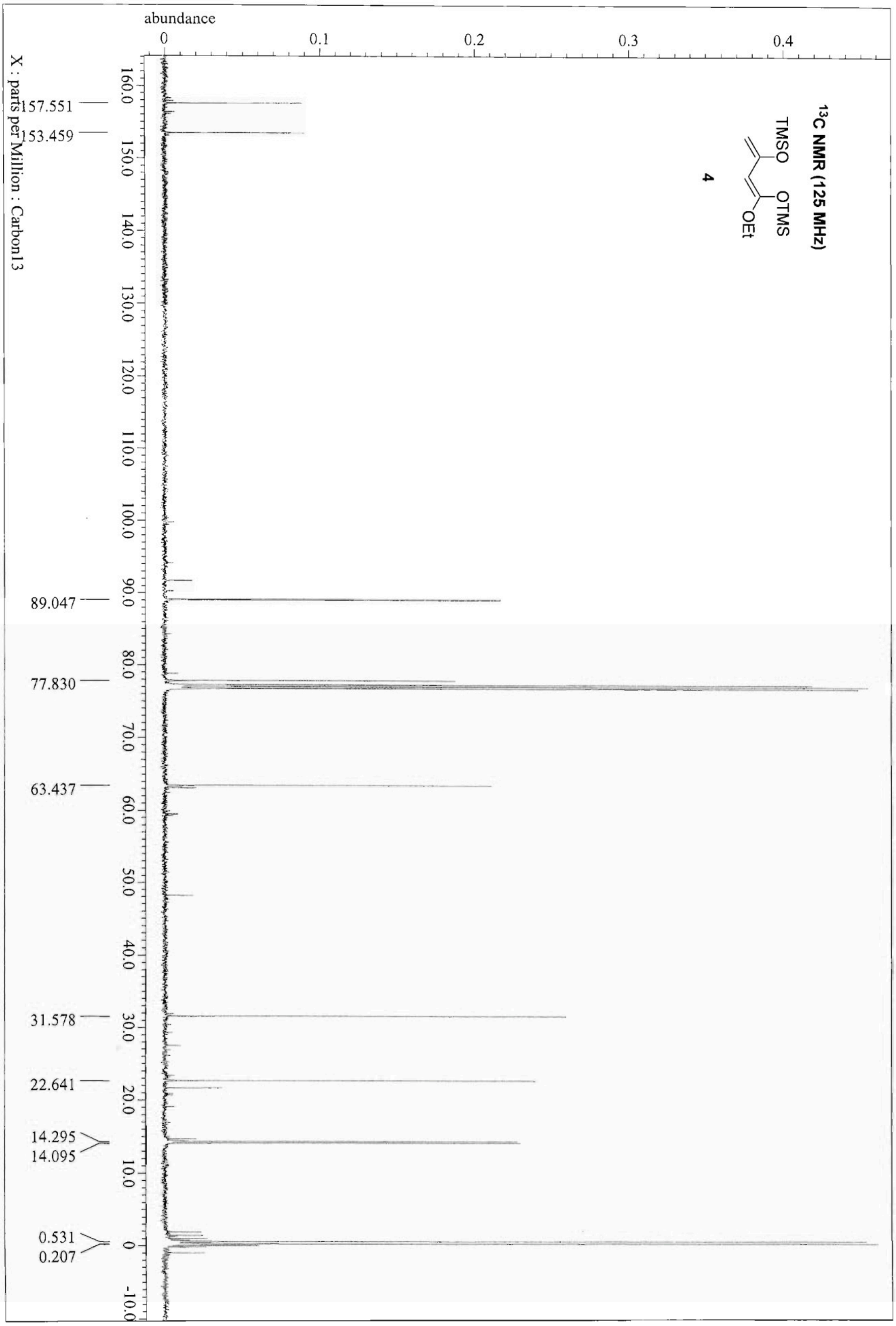


**Figure S4.** $^{13}$C-NMR chart of **4**.

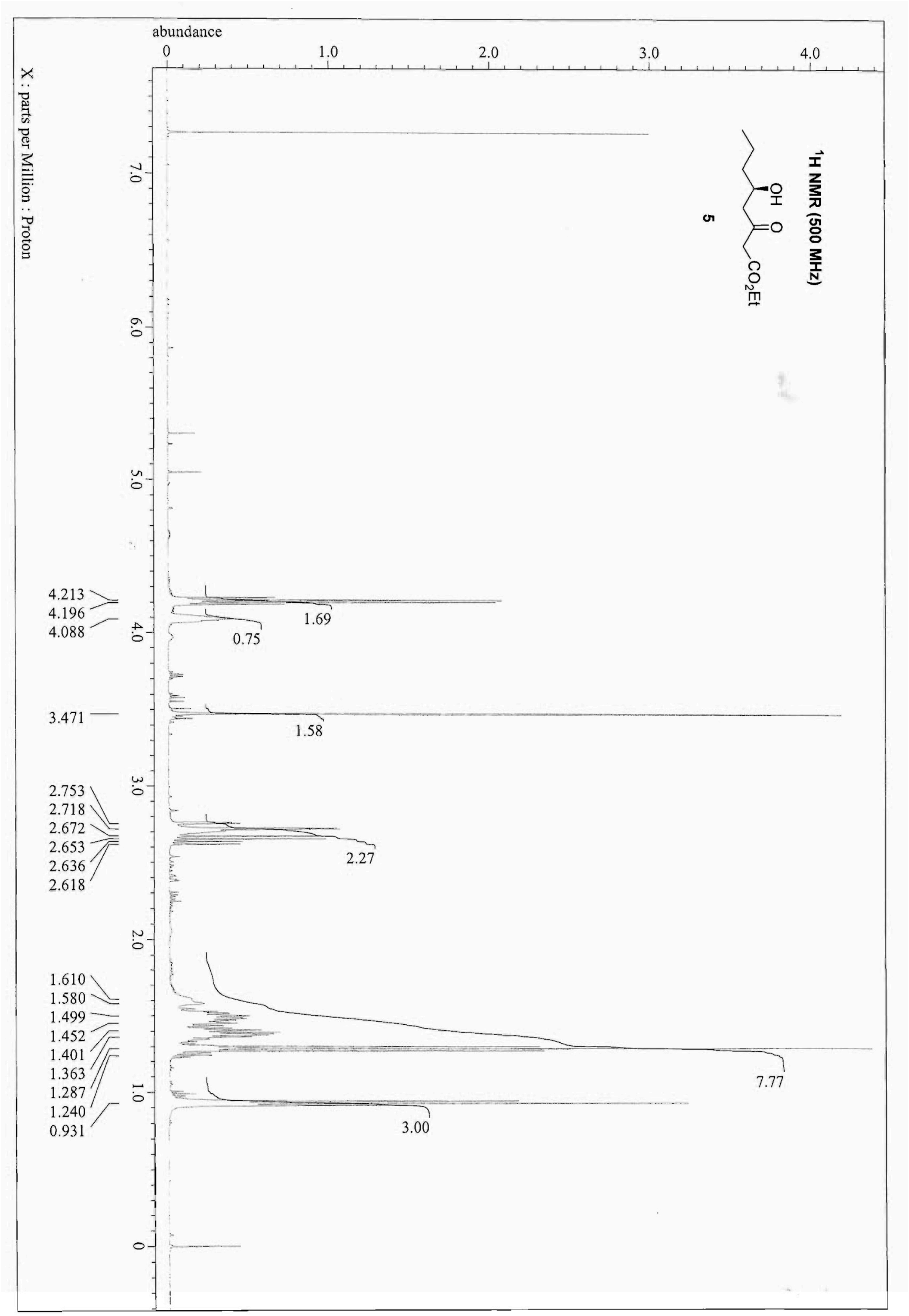


**Figure S5.** $^{1}$H-NMR chart of **5**.

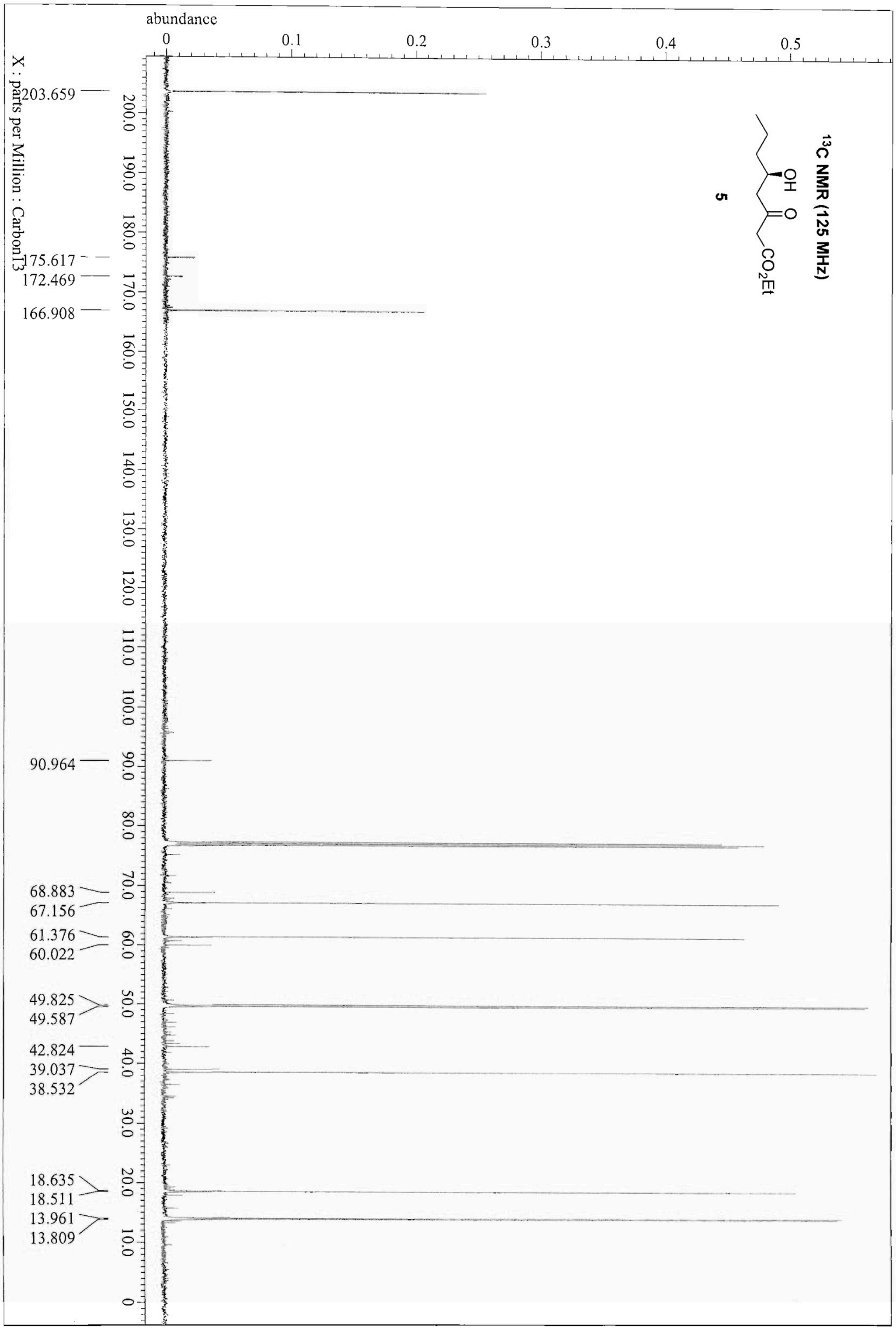


**Figure S6.** $^{13}$C-NMR chart of **5**.

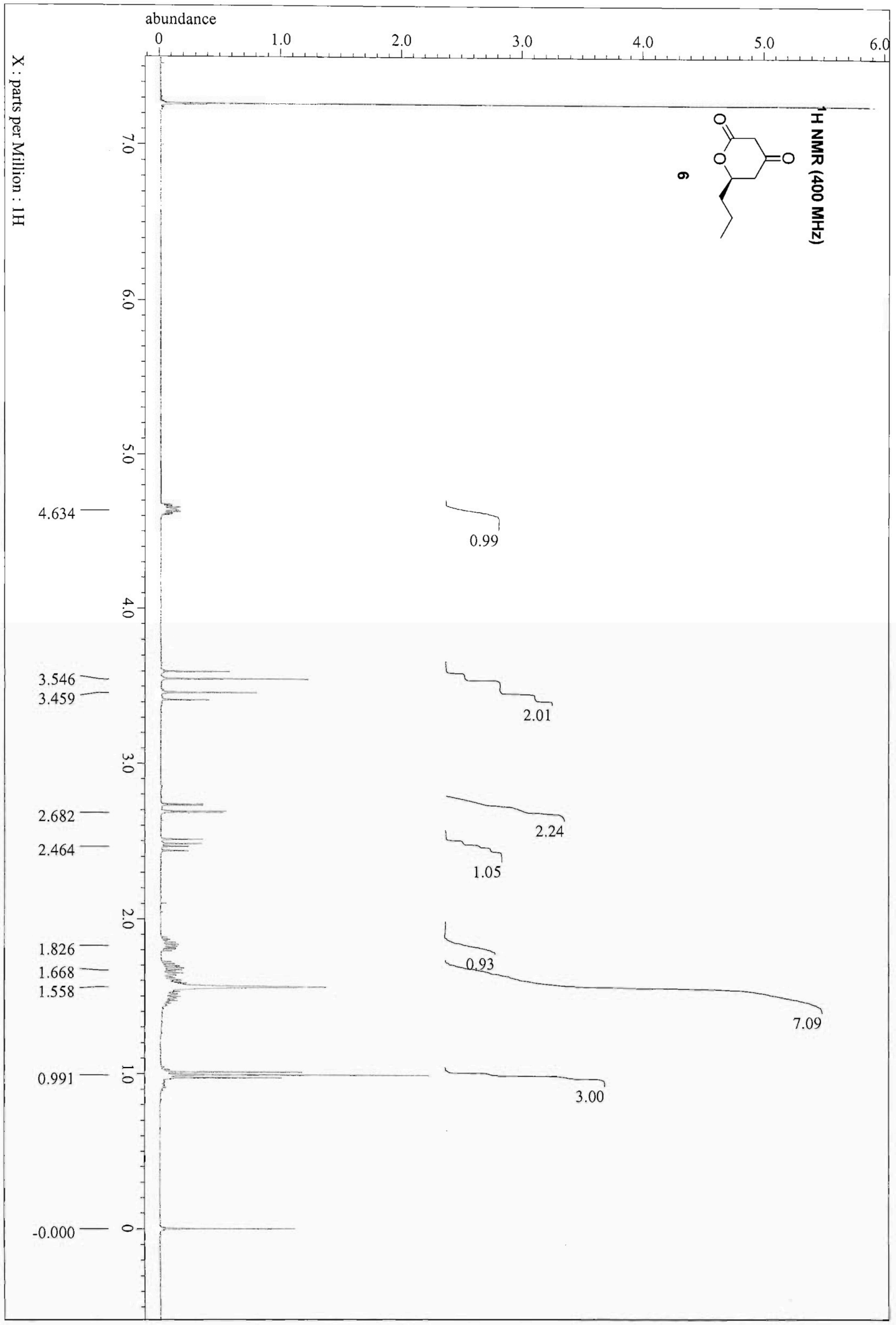


**Figure S7.** ¹H-NMR chart of **6**.

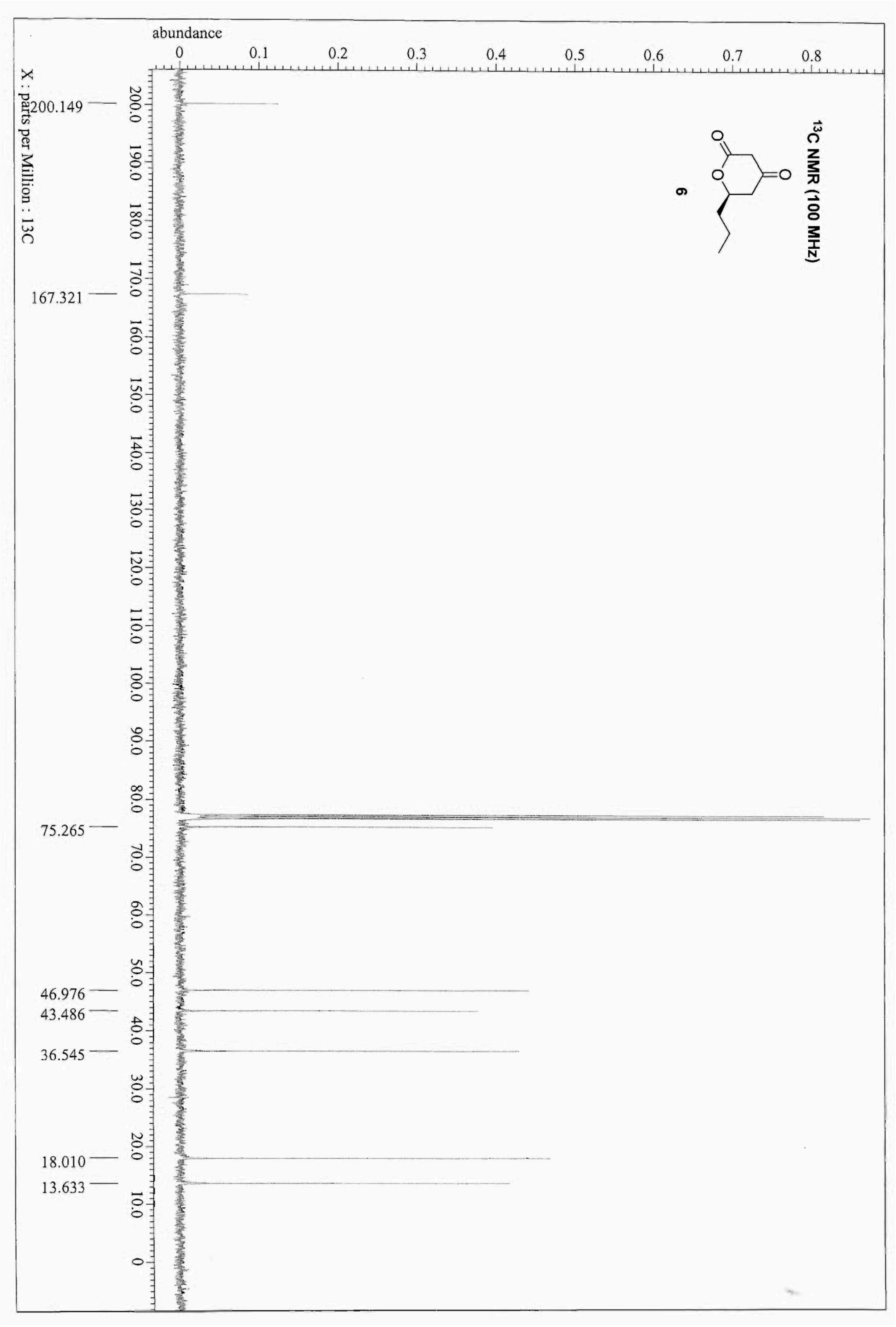


**Figure S8.** $^{13}$C-NMR chart of **6**.

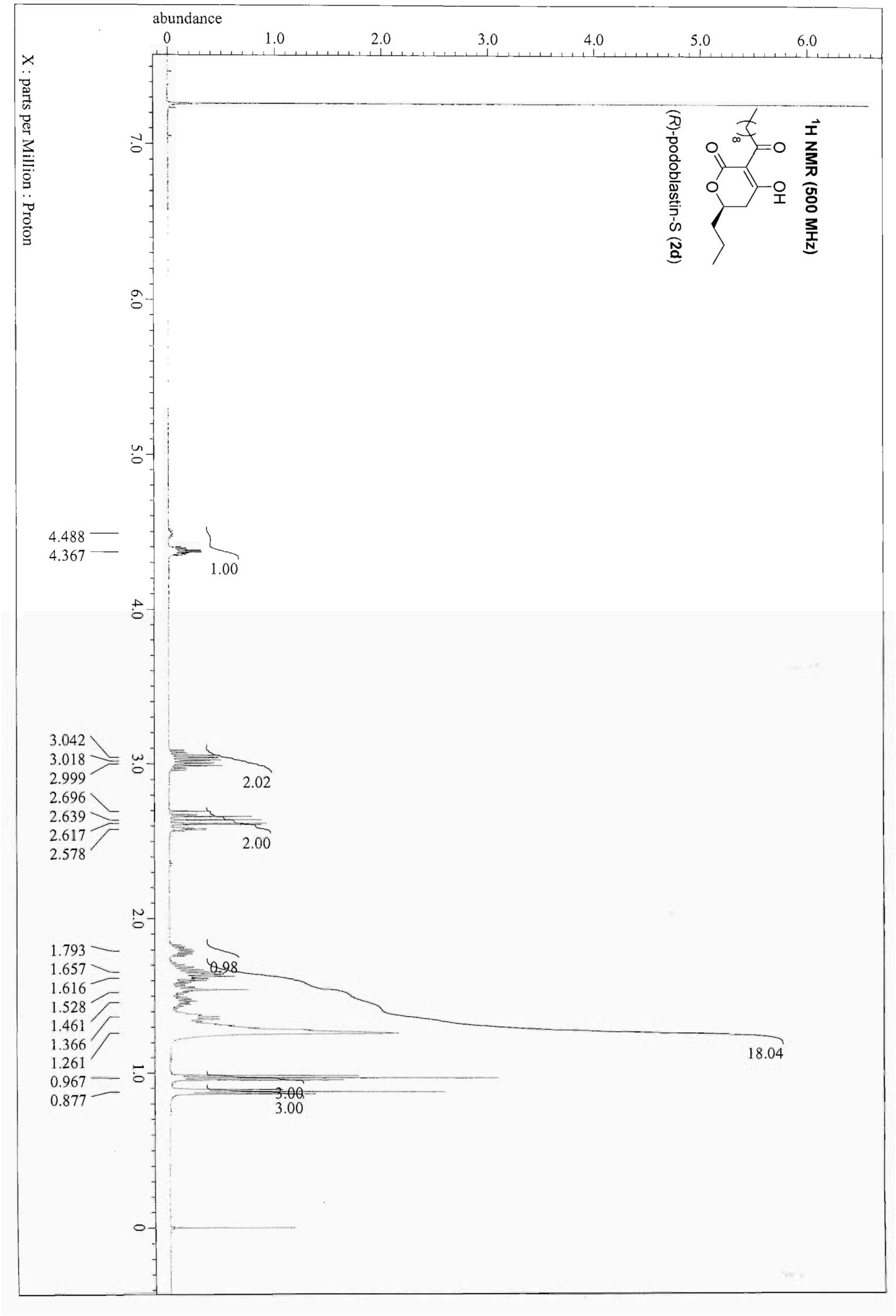


**Figure S9.** $^{1}$H-NMR chart of **2d**.

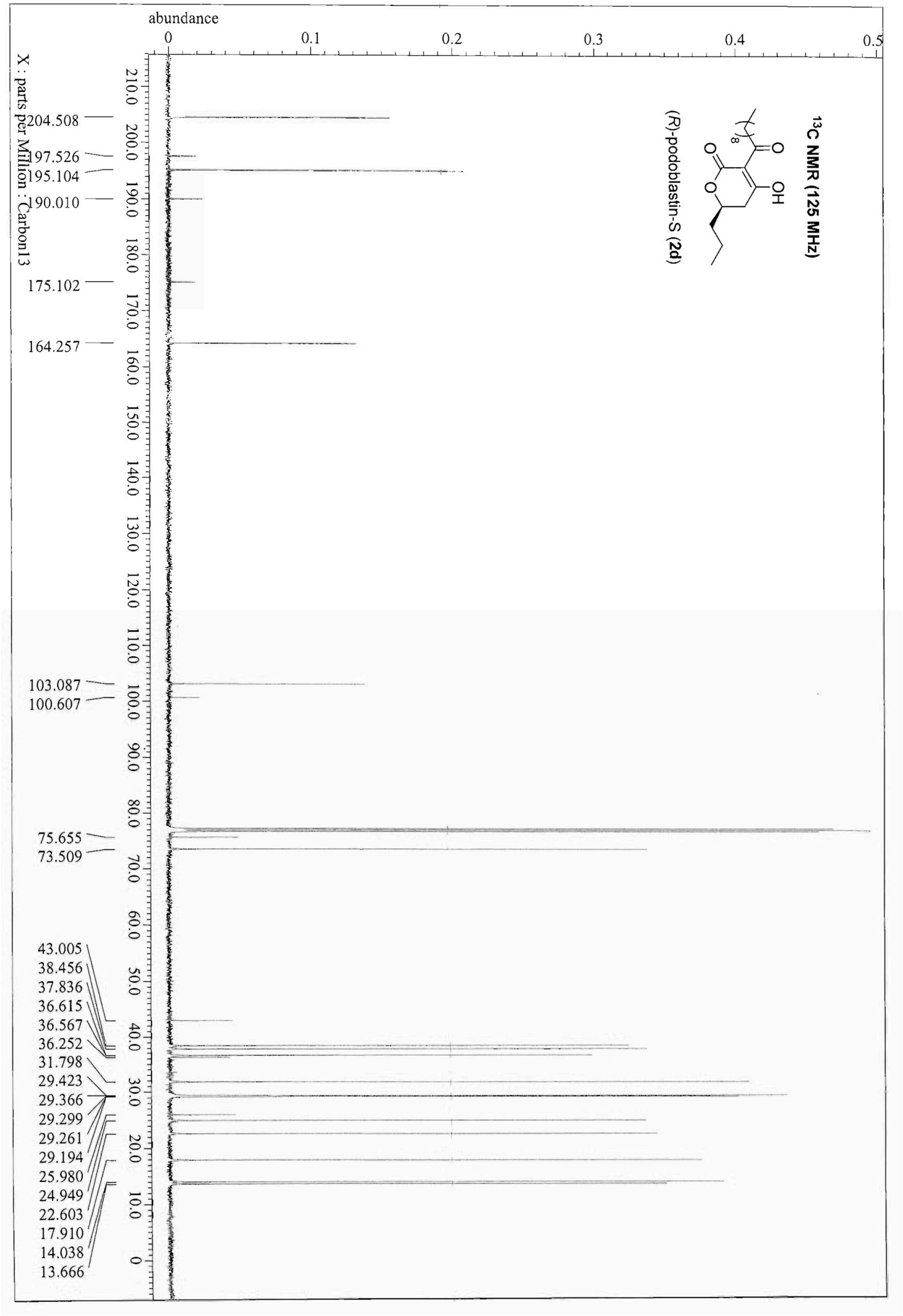


**Figure S10.** $^{13}C$-NMR chart of **2d**.

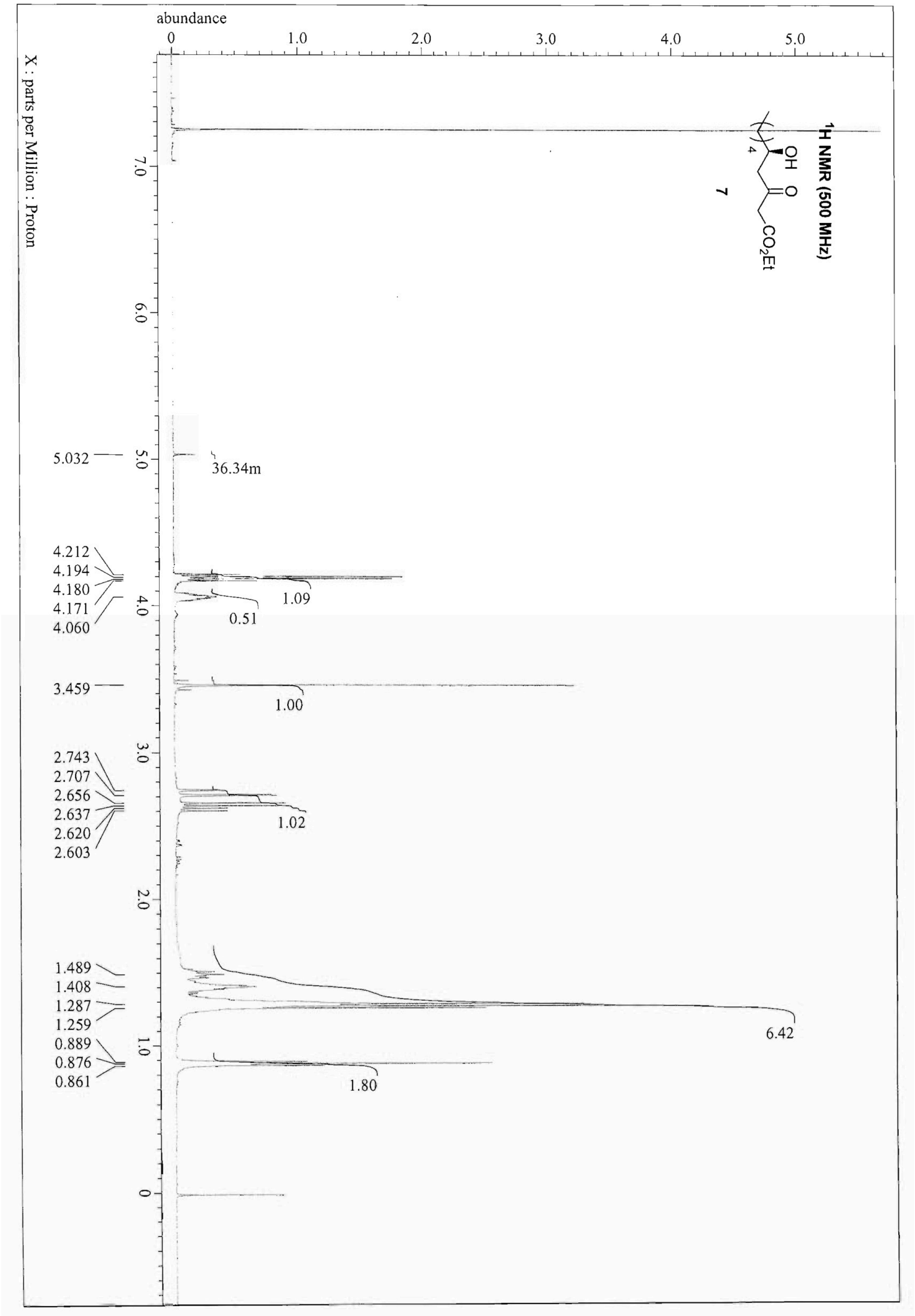


**Figure S11.** $^{1}$H-NMR chart of **7**.

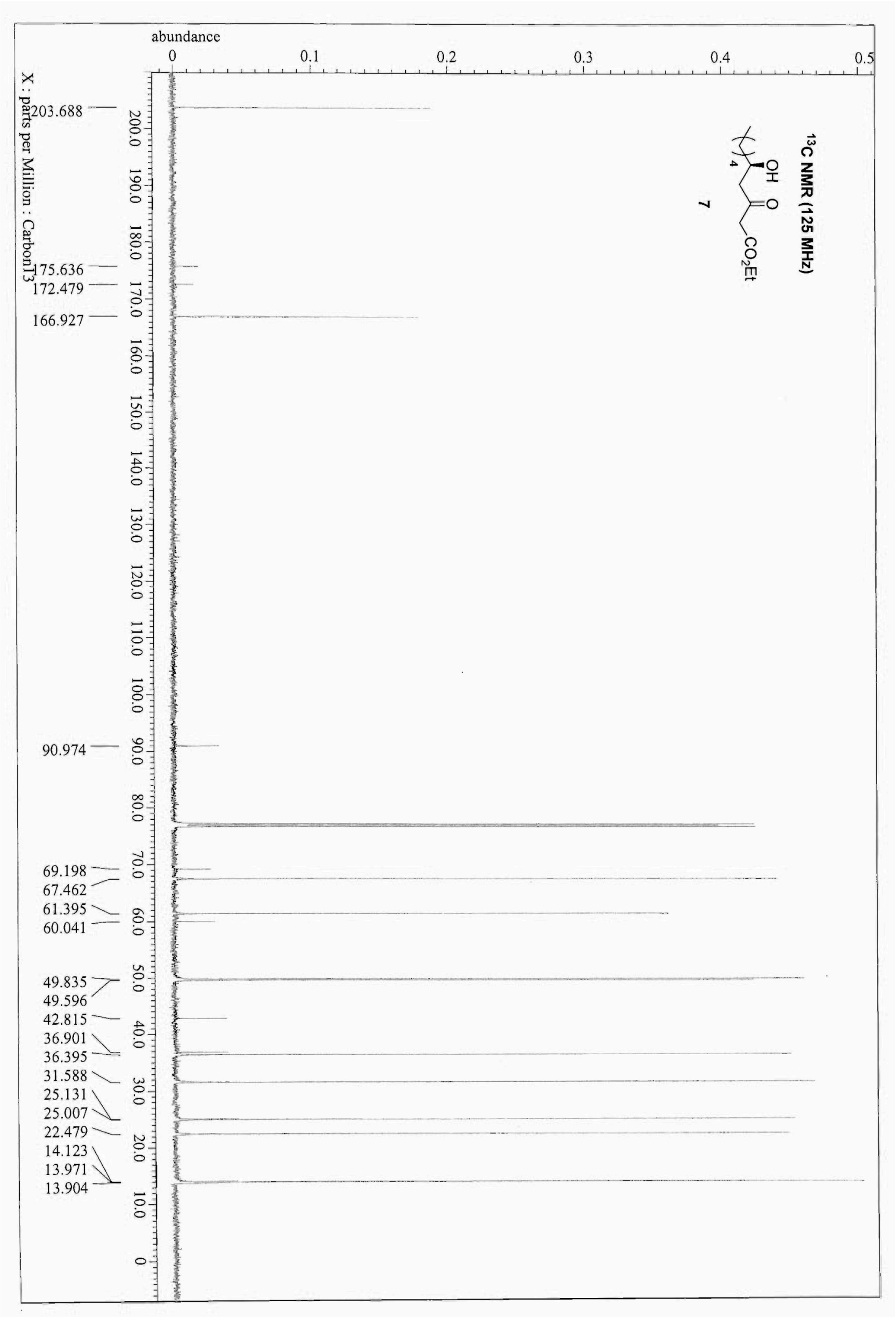


**Figure S12.** $^{13}$C-NMR chart of **7**.

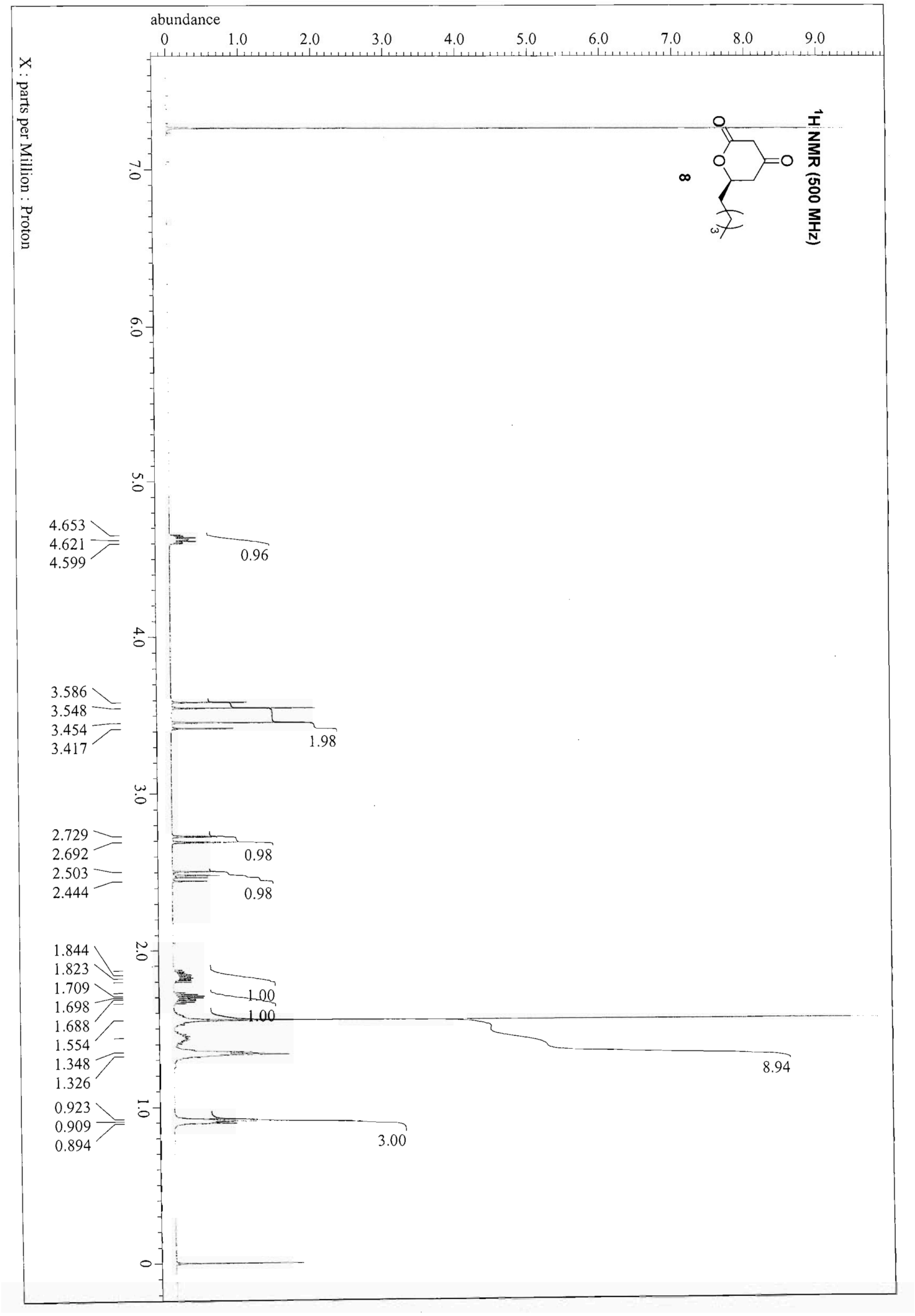


**Figure S13.** $^{1}$H-NMR chart of **8**.

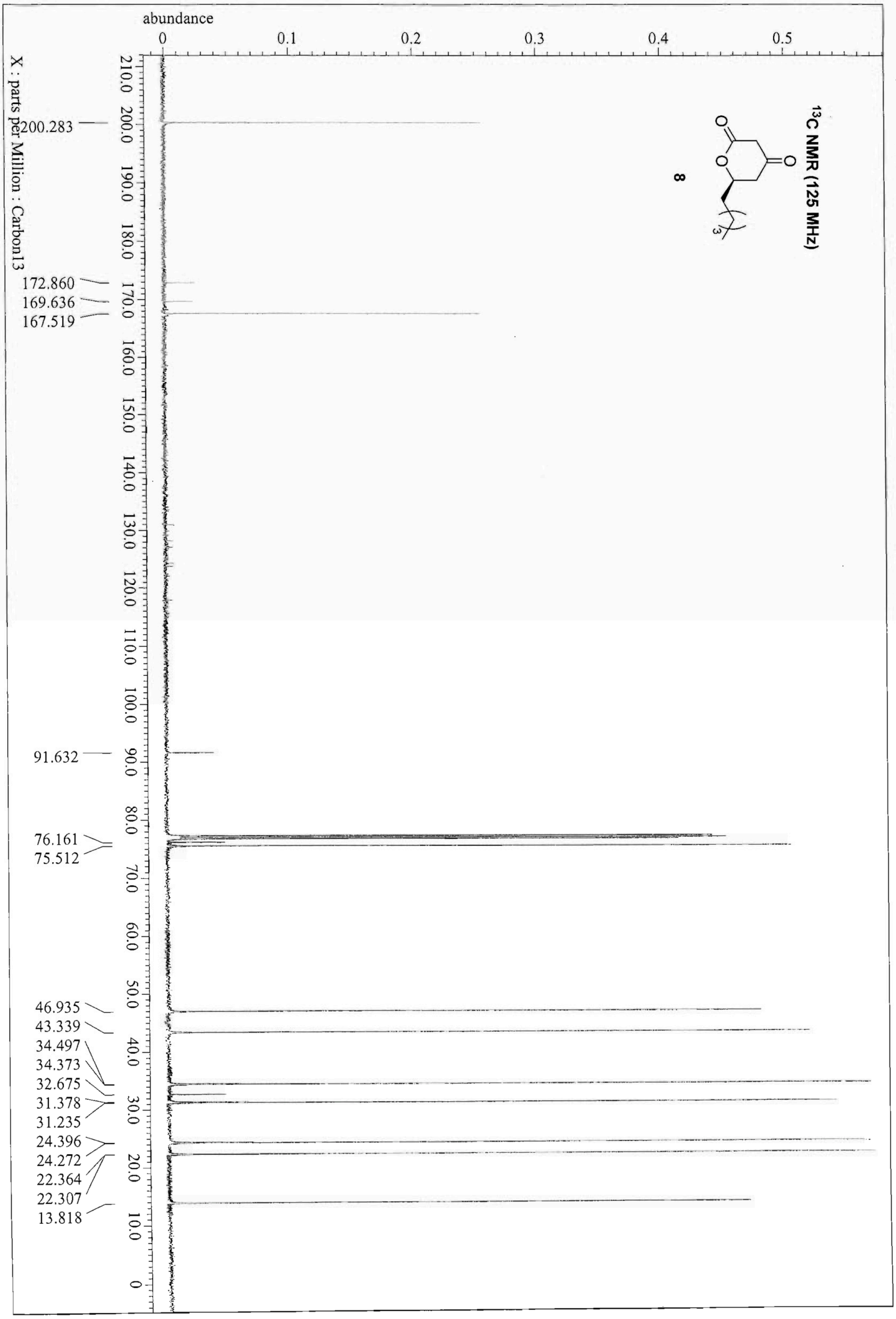


**Figure S14.** $^{13}$C-NMR chart of **8**.

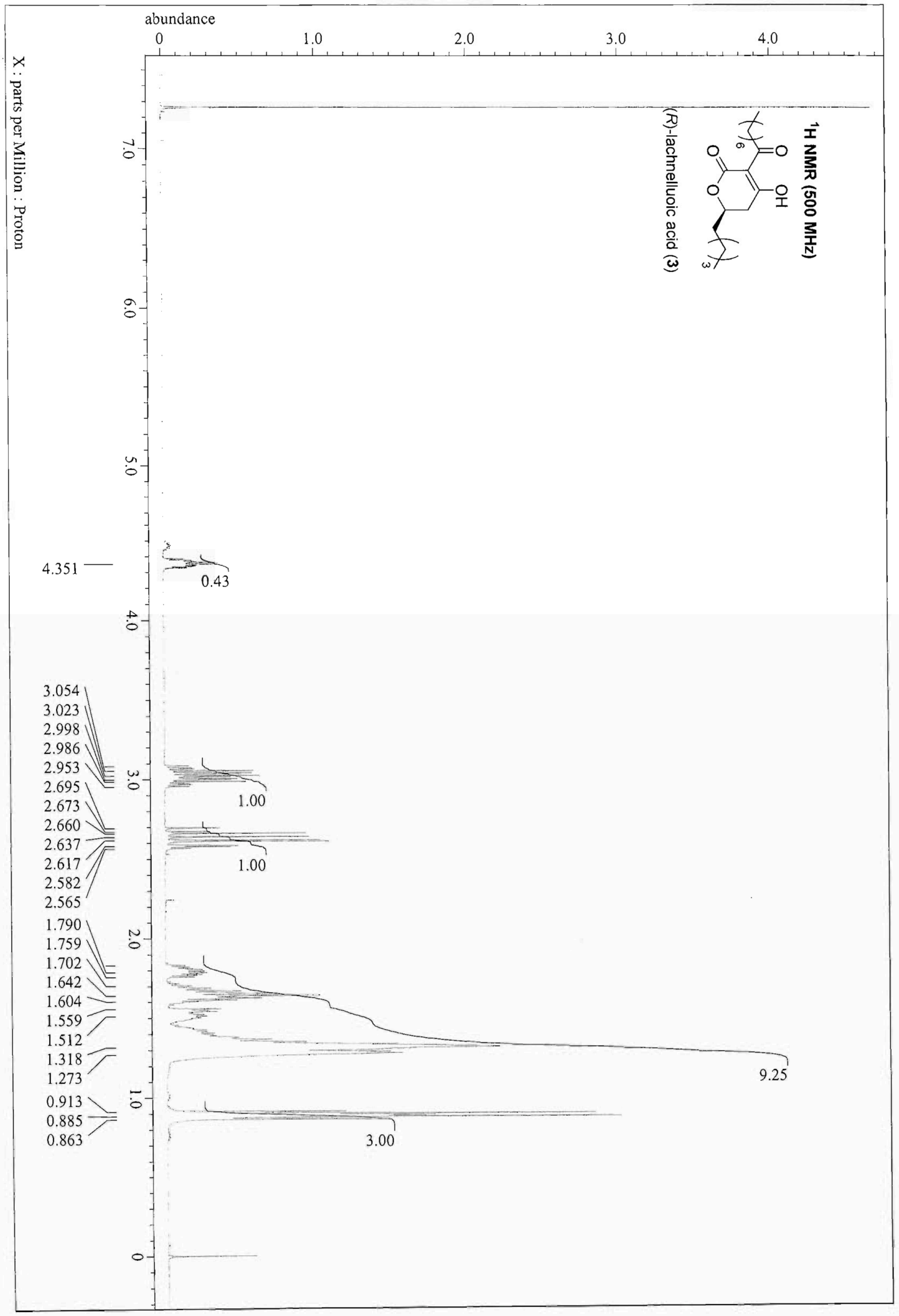


**Figure S15.** $^1$H-NMR chart of **3**.

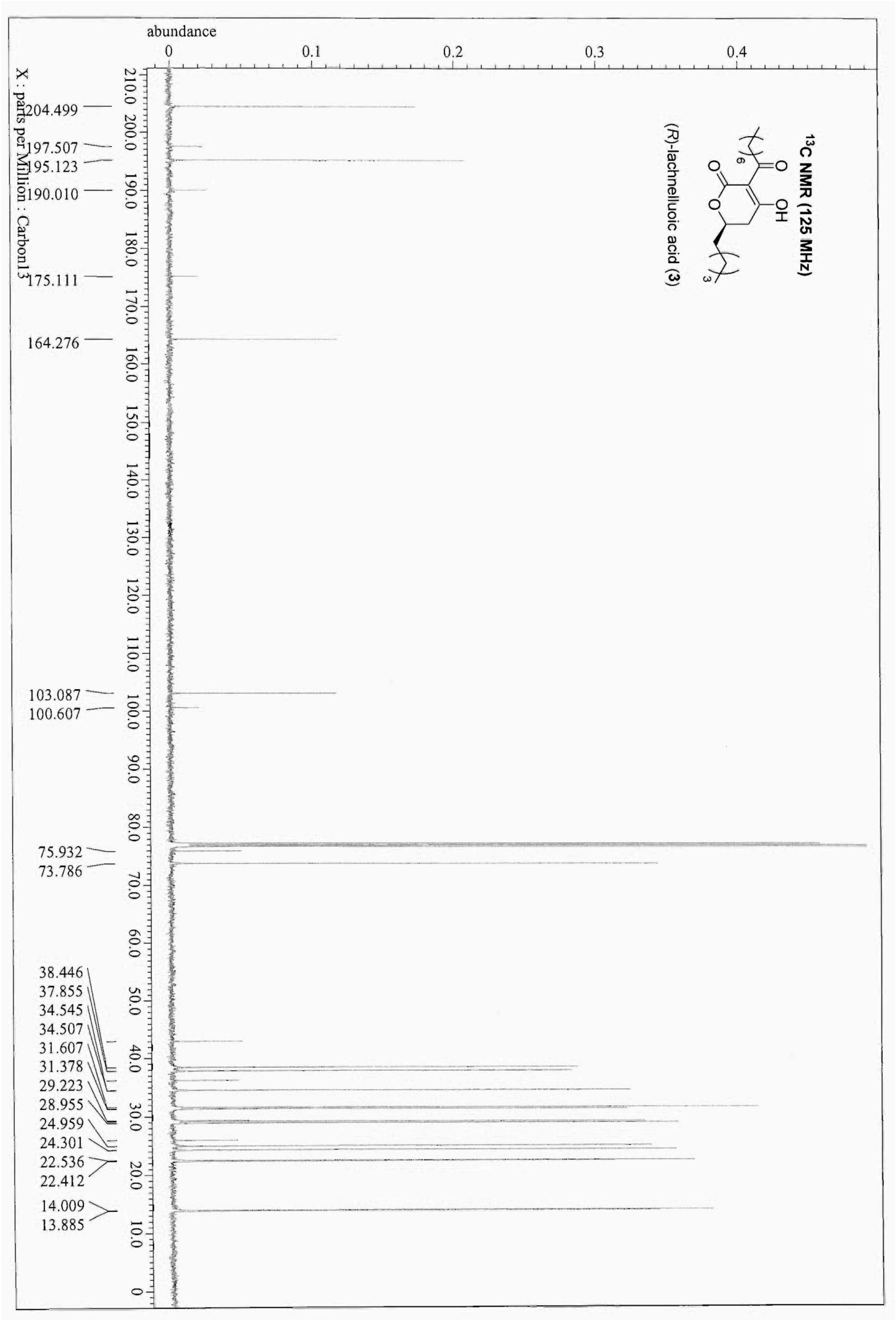


**Figure S16.** $^{13}$C-NMR chart of **3**.
